# Application of EEG and Interactive Evolutionary Design Method in Cultural and Creative Product Design

**DOI:** 10.1155/2019/1860921

**Published:** 2019-01-13

**Authors:** Li Deng, Guohua Wang

**Affiliations:** ^1^Key Laboratory of Oil and Gas Equipment, Ministry of Education, Southwest Petroleum University, Chengdu 610500, China; ^2^School of Mechatronic Engineering, Southwest Petroleum University, Chengdu 610500, China; ^3^State Key Laboratory of Oil and Gas Reservoir Geology and Exploitation, Southwest Petroleum University, Chengdu 610500, China

## Abstract

In order to design a cultural and creative product that matched the target image, this paper proposed to use EEG, interactive genetic algorithm (IGA), and back propagation neural network (BPNN) to analyze the users' image preferences. Firstly, the pictures of cultural elements were grouped according to the pleasantness value and emotional state by PAD emotion scale, and the brain waves induced by the pictures of cultural elements with different pleasure degree were recorded by electroencephalograph. Then, the preference of cultural elements was obtained according to the theory of frontal alpha asymmetry. Secondly, the semantic difference method was used to carry out questionnaire survey to users, and the factor analysis method was used to statistically analyze the survey results to extract the perceptual image semantics of users for cultural and creative products. Thirdly, an interactive evolutionary design system based on IGA and BPNN was constructed. According to the cultural elements preferred by users, the designer designed the initial set of morphological characteristics, and the fitness value was determined according to the degree of user preference for the image semantics. Meanwhile, in order to reduce the fatigue caused by users' interaction evaluation, BPNN was introduced to simulate artificial evaluation. Finally, the proposed method was verified by the practice of flavoring bottle design. User preference requirement could be used as feedback information to help designers understand users' design emotional need and generate design schemes that satisfied the users' perceptual image.

## 1. Introduction

With the advent of experience economy, it is a key link of innovative product design to obtain user preferences quickly and accurately [1]. Therefore, this paper attempted to use EEG technology and interactive evolutionary design method to analyze users' emotional experience. Based on user preferences to conduct product shape design and the users participated in cultural and creative product design by computer-aided design, which could directly and effectively help designers to obtain the information of design requirements.

In the process of cultural and creative product design, many scholars have studied the extraction of cultural genes and design elements. For example, Gou et al. [2] extracted the cultural genes of Banpo painted pottery based on genetic theory. Wang et al. [3] extracted the form, color, and connotation factors contained in traditional culture. Zhu and Luo [4] interpreted and excavated cultural elements from four dimensions: semantic, syntactic, contextual, and pragmatic. Chai et al. [5] analyzed the impact of different cultural elements on consumer satisfaction using continuous fuzzy Kano model. Liu et al. [6] studied the extraction of color features from the traditional pattern library and recommended color scheme that best reflected the original features of culture for designers. Luo and Dong [7] introduced the concept of “ontology” in knowledge engineering and developed a management system of cultural artifacts knowledge for cultural creative design. The cultural and creative products designed by the above methods could reflect the traditional cultural elements, but whether the products met the emotional need of users required further research.

When a user is observing a product, the external visual stimulation will induce the changes of EEG. Analyzing EEG signals can accurately and objectively measure users' perception, preference and emotion, and obtain users' psychological needs. At present, EEG technology has been widely applied in the field of industrial design, such as commercial advertising design [8], seat design [9], interface design [10], comfort evaluation [11], and so on. In the field of emotional measurement, Li et al. [12] studied the influence of apple tree's leaves and flowers on human brain waves and found that different visual stimuli would induce different emotions. Zhuang [13] selected two shopping websites with large differences in appearance and usability as the object of study. Then, taking EEG and eye-movement index as independent variables, an emotional measurement model was constructed by partial least square regression method. Aiming at the scheme selection problem of automobile industry design, Tang et al. [14] proposed to objectively evaluate the user experience through EEG data. Therefore, this paper could use the above research for reference in cultural and creative product design, and use EEG technology for quantitative research to help designers to develop products that meet users' emotional need.

In the traditional cultural and creative product shape design, designers use personal experience and subjective speculation to obtain users' emotional need. Because of the lack of scientific and objective evaluation mechanism, users' image preference cannot be reflected in product design accurately and quickly. It is a research hotspot to integrate decision makers' advantage into evolutionary design methods. Gong et al. [15] presented a novel evolutionary algorithm based on the theory of preference polyhedron that interacted with a decision maker during the optimization process to obtain the most preferred solution. In many algorithms, IGA is effective method of solving optimization problems with implicit criteria by incorporating a user's intelligent evaluation into traditional evolution mechanisms [16]. In IGA, decision makers give the individual fitness evaluation by interactive means. It has very high application value and extensive practical significance in the field of artistic creation, design, and other areas of that bias towards human subjective feeling. IGA has been applied to clothing design [17], chair design [18], building design [19], logo design [20], product color planning [21], personalized search [22], and so on. However, IGA also has some deficiencies. In the process of user evaluation, users are prone to fatigue. Therefore, how to reduce the fatigue caused by users' interaction evaluation is the research point of many scholars. For example, Xu and Sun [23] put forward a product modeling design method based on orthogonal-interactive genetic algorithm. Gong et al. [24] introduced the idea of stratification into interactive evolutionary computation and proposed hierarchical interactive evolutionary computation. Sun et al. [25] proposed a new surrogate-assisted IGA, where the uncertainty in subjective fitness evaluations was exploited both in training the surrogates and in managing surrogates.

In order to reduce the users' fatigue during the interaction evaluation in IGA, this paper introduced neural network to assist IGA. Neural network is a nonlinear algorithm, which is often used to establish the relationship between complex input and output variables, and is successfully applied to the field of product shape design by perceptual image. Using the method of fuzzy neural network, Hsiao and Tsai [26] constructed the correspondence relationship between the modeling data of conceptual product and the perceptual image vocabulary. Diego-Mas and Alcaide-Marzal [27] proposed a consumer emotional response model based on neural network. Through experimental research on mobile phones, Yeh and Lin [28] established an artificial neural network model between product image and consumer's perceptual image based on the concept of Kansei engineering. In view of the feasibility of neural network in Kansei engineering design, this paper combined BPNN to simulate user evaluation to realize automatic solution of the scheme.

This paper took the flavoring bottle design as an example to explore the innovative shape design scheme. Based on the cognition of users and designers, the product scheme was optimized by combining IGA and BPNN, to reflect the users' emotional need in product design accurately.

## 2. Outline of the Proposed Method

**Figure 1:**
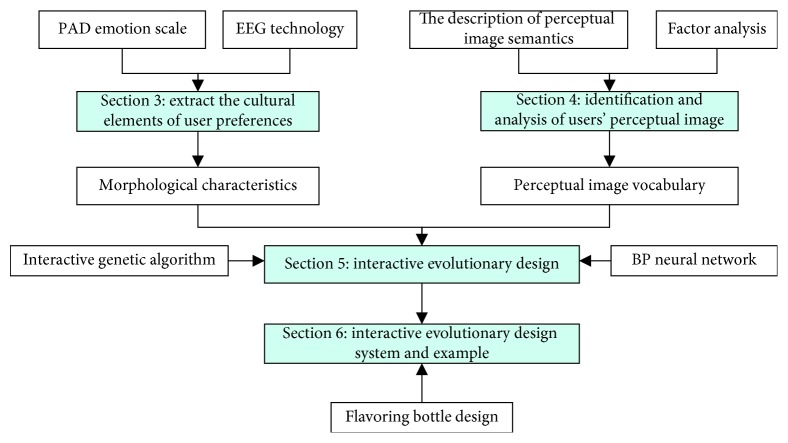
A summary of the proposed method.

As shown in Figure 1, this paper first extracts the cultural elements preferred by users and sets it as the source of morphological characteristics for subsequent evolutionary design (see Section 3). Secondly, we extract the users' perceptual image for cultural and creative products, which will be set as the target image of subsequent evolutionary design (see Section 4). On this basis, interactive evolutionary design is carried out. IGA is used to achieve the product shape evolution, and the fatigue problem of interactive evaluation is solved by combining with BPNN (see Section 5). Finally, the process is verified by the example of flavoring bottle design (see Section 6).

## 3. Extract the Cultural Elements of User Preferences by EEG

### 3.1. Application Principle of EEG Technology

According to the different frequencies, EEG activity is divided into *δ* wave (<3 Hz), *θ* wave (4–8 Hz), *α* wave (8–13 Hz), *β* wave (13–30 Hz), and *γ* wave (30–45 Hz). Each frequency band corresponds to different cognitive characteristics. Studies have shown that the different product designs can be compared using the frontal alpha asymmetry (FAA), and the activity of left frontal lobe is often used as an indicator of pleasure or liking [29]. The “cerebral hemispheric potency hypothesis” suggests that the left frontal region mainly deals with positive emotions, while the right frontal region mainly deals with negative emotions [30, 31]. In EEG, the power of *α* wave in the left frontal lobe decreases and the activity of left hemisphere increases in the positive mood state; the power of *α* wave in the right frontal lobe decreases, and the activity of right hemisphere increases in the negative mood state. The power size is inversely proportional to the activity of brain, and the declining power of *α* wave in the left frontal lobe indicates that the subjects are in a more pleasant state. The frontal *α* wave can be used as an indicator of the relationship between product experience and user emotion [13]. Therefore, this paper used the power of frontal *α* wave to analyze user preferences for different cultural elements of “Shu culture”.

### 3.2. Obtain Visual Stimulation Materials by PAD Emotion Measurement

#### 3.2.1. PAD Emotion Scale

PAD emotion scale can accurately evaluate the emotional state from three dimensions of pleasure, arousal, and dominance, and it has good structural validity. Therefore, this paper used PAD emotion scale to measure users' emotion for different cultural elements [32]. *P* represents pleasure-displeasure, *A* represents arousal-nonarousal, and *D* represents dominance-submissiveness [33]. According to the above three dimensions, the emotional state can be divided into eight categories: (1)+*P* + *A* + *D* (happy), (2)−*P* − *A* − *D* (boring), (3)+*P* + *A* − *D* (dependent), (4)−*P* − *A* + *D* (disdainful), (5)+*P* − *A* + *D* (relaxed), (6)−*P* + *A* − *D* (anxious), (7)+*P* − *A* − *D* (gentle), and (8)−*P* + *A* + *D* (hostile).

**Figure 2:**
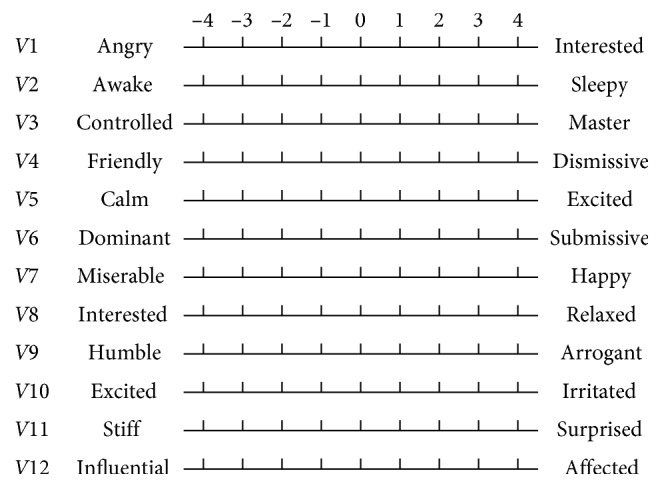
PAD emotion scale.

PAD emotion scale consists of twelve pairs of adjectives representing different emotional state (see Figure 2) and each four pairs of adjectives constitute a dimension. Every pair of adjectives expresses the opposite emotion on the dimension that they belong, and the emotional values on the other two dimensions are almost the same. The nine-point semantic differential scale is used to quantify these emotions. For example, *V1*: angry-interested, and the score is between −4 and 4. The subjects score according to their emotional intensity. From left to right, the more left the score is, the more “angry” it is. The more right the score is, the more “interested” it is. The relationship between the scores of pleasure (*P*), arousal (*A*), dominance (*D*), and the scores of the above twelve pairs of adjectives is shown by formulas (1)–(3). The score of each dimension is the average score of the four groups of adjectives. The higher the PAD score, the higher the pleasure, arousal, and dominance, respectively.

(1) $$\begin{array}{c} P=\frac{\left(V1-V4+V7-V10\right)}{4}, \end{array}$$

(2) $$\begin{array}{c} A=\frac{\left(-V2+V5-V8+V11\right)}{4}, \end{array}$$

(3) $$\begin{array}{c} D=\frac{\left(V3-V6+V9-V12\right)}{4}. \end{array}$$

#### 3.2.2. Emotion Test

**Figure 3:**
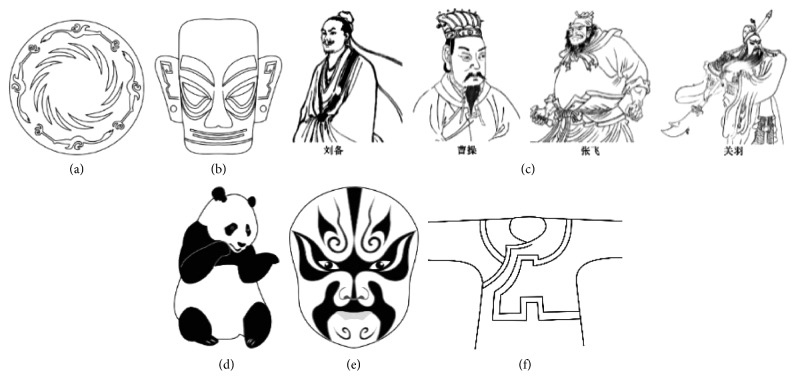
The samples of cultural elements from “Shu culture”. (a) Jinsha. (b) Sanxingdu. (c) Three kingdoms personages. (d) Panda. (e) Sichuan opera mask. (f) National costume.

As shown in Figure 3, six representative cultural elements from “Shu culture” have been selected as picture samples, and every picture is numbered. PAD emotion scale is shown in Figure 2. Ten (five male and five female) industrial design students participated in the emotion test. For the six pictures, every subject was asked to score the twelve items in the scale of Figure 2, respectively, with the score range of −4 to 4.

According to the corresponding dimension of adjectives, the emotional values of the three dimensions were calculated by formulas (1)–(3), and the emotional classification was made according to the positive and negative situation of the values (Table 1).

According to the value of pleasure (*P*) and the category of emotional state in Table 1, the six picture samples were divided into three groups: like, general, and dislike (see Table 2). Three groups of picture samples with different emotional states were set as the visual stimulus materials for subsequent EEG experiments to induce the subjects to have different emotional experiences.

### 3.3. EEG Experiment Process

1. Subject

Twenty students in the department of industrial design were invited as subjects. They were required to have a good rest before the experiment, without taking medicine and drinking. A small gift was given as a reward after the experiment.

(2) Experimental environment and equipment

The experiment was carried out in the human-machine laboratory, the indoor temperature was suitable, and there was no noise interference. The experiment used the EEG equipment from German Brain Products, including amplifier, electrode cap, conductive paste and other hardware, stimulus presentation software E-Prime, data acquisition software Brain Vison Recorder, and analysis and processing software Brain Vision Analyzer 2.1.

(3) EEG recording

**Figure 4:**
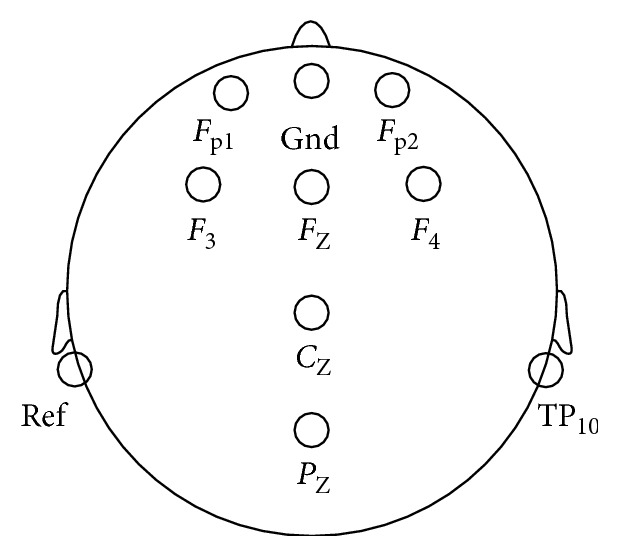
Position distribution of electrode.

The cerebral cortex is mainly divided into frontal lobe, parietal lobe, occipital lobe, and temporal lobe. Each brain region contains a large number of neurons, taking on different tasks. The frontal lobe is mainly related to thinking, emotion, planning, and needs. Therefore, the frontal lobe was selected as the study object in this experiment. The EEG data of channels such as *F*_P1_ (left frontal pole), *F*_P2_ (right frontal pole), *F*_3_ (left frontal), *F*_4_ (right frontal), *F*_Z_ (middle frontal line), *C*_Z_ (central midline), and *P*_Z_ (top midline) were recorded. Electrode arrangement is shown in Figure 4.

(4) Experimental procedures

**Figure 5:**
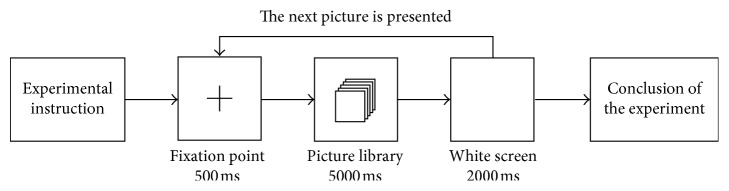
Experimental flow chart.

The six picture samples were processed by the Photoshop software with uniform size and white background and the cultural elements expressed by black lines to exclude the influence of irrelevant factors such as color. The experimental process was presented by E-prime software. As shown in Figure 5, first, the experimental instructions inform the subjects to perceive the whole picture of “Shu culture element” that appears. Then, the center of computer screen shows the cross fork 500 ms that guides the subjects to be noticed. Next, a picture of “Shu culture element” is presented 5000 ms. At last, when the picture disappears, a white screen is presented 2000 ms, so as to eliminate the visual residue of the subjects. This process repeated until all the pictures were presented one by one, and the EEG data were stopped recording.

(5) Experimental steps

First, prepare for the work. Before the formal experiment, in order to eliminate the nervousness, the subjects should be informed of the experimental purpose, experimental process, and the matters needing attention in the experimental process. Secondly, the electrode cap was worn on the subject's head, and it was placed in the way from front to back. The placement of the electrode is shown in Figure 4. An appropriate amount of conductive paste was injected to ensure that the impedance of all leads was reduced below the required value. The subjects adjusted comfortable sitting posture, the eyes were about 50 cm away from the computer screen, and they could not move their body parts during the experiment. Then, the experiment was officially started. Brain Vison Recorder was used to record the subjects' brain waves during the experiment.

### 3.4. Experimental Data Analysis

**Figure 6:**
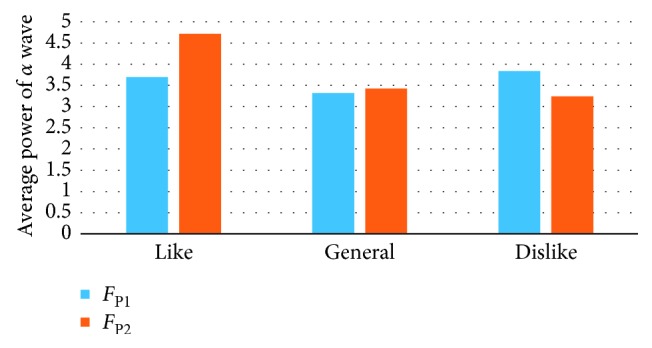
The average power of *α* wave at *F*_P1_ and *F*_P2_ electrodes.

**Figure 7:**
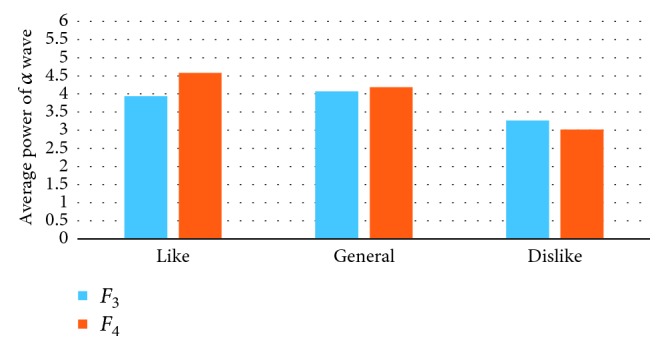
The average power of *α* wave at *F*_3_ and *F*_4_ electrodes.

**Figure 8:**
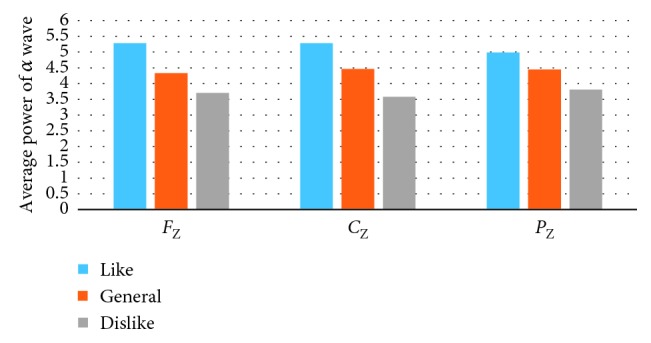
The average power of *α* wave at *F*_Z_, *C*_Z_, and *F*_P2_ electrodes.

The EEG power spectrum of the collected data was analyzed by Brain Vision Analyzer 2.1. The EEG signals evoked by the picture presentation 5000 ms were extracted. After digital filtering, segmentation, and operation, EEG data were quantitatively analyzed. Then, the average power of *α* wave (8∼13 Hz) at the electrodes of *F*_P1_, *F*_P2_, *F*_3_, *F*_4_, *F*_Z_, *C*_Z_, and *P*_Z_ was obtained. Figures 6–8 show the average power of *α* wave stimulated by the pictures with different preference. Under the stimulation of the favorite pictures, the average power of *α* wave at *F*_P1_ (left frontal pole) and *F*_3_ (left frontal) is lower than that at *F*_P2_ (right frontal pole) and *F*_4_ (right frontal). Under the stimulation of the disliked pictures, the average power of the *α* wave at *F*_P2_ (right frontal pole) is lower than that at *F*_P1_ (left frontal pole), and the average power of *α* wave at *F*_3_ (left frontal) and *F*_4_ (right frontal) is not significantly different. Under the stimulation of the general pictures, there is no significant difference between the average power of *α* wave at *F*_P1_ (left frontal pole), *F*_3_ (left frontal) and that at *F*_P2_ (right frontal pole), *F*_4_ (right frontal). In the central region (*F*_Z_, *C*_Z_, *P*_Z_), the average power of *α* wave under the stimulation of the favorite pictures is significantly higher than that under the stimulation of the disliked pictures.

To sum up, under the stimulation of the favorite picture samples, the average power of *α* wave in the left frontal pole and left frontal decreased, indicating that this group of pictures induced the subjects to have pleasant emotions. In contrast, under the stimulation of the disliked picture samples, the average power of *α* wave in right frontal pole decreased, indicating that this group of pictures induced the subjects to have unpleasant emotions. The experimental results were consistent with the “cerebral hemispheric potency hypothesis.” In addition, the power of *α* wave in the left and right frontal poles under the stimulation of each picture sample is analyzed in Table 3. It was found that this rule was also applicable to single-picture sample. Therefore, the asymmetry of the power of *α* wave in the left and right frontal poles could be used to judge the emotional state of the subject, which would help the designers to obtain user preference information through user emotion. From the data in Table 3, it can be seen that the subjects are inclined to the cultural element of national costume in cultural and creative product design of “Shu culture”.

## 4. The Investigation and Extraction of Users' Perceptual Image

### 4.1. The Description and Evaluation of Perceptual Image Semantics

According to Kansei engineering, the following steps were used to obtain the image semantics of users on cultural and creative products:

The first was to select image vocabulary. The emotional preference for cultural and creative products could be abstracted as adjectives, and the selection of representative adjectives would affect the accuracy and authenticity of experimental results. The perceptual image vocabularies were used to describe the consumers' cognitive and visual feelings about cultural and creative products. These vocabularies constituted the semantic set *C* of perceptual image:

 
*C* = {*C*1 Unadorned, *C*2 Fashionable, *C*3 Succinct……*Cn* Advanced}.

The second was to use pictures to arouse the emotional image feelings of different dimensions of vocabularies and to strengthen the users' perception of image scale.

Finally, the products and the perceptual image adjectives were evaluated interactively. The scale was designed according to the fuzzy statement “the perceptual cognitive preference *C*_*i*_ for cultural products *W*_*m*_ is *V*_*j*_.” The five-point scale was used to distinguish the users' preference degree for perceptual image. The users' score constituted the set of state degree *V*:

   *V* = {very not 1 not 2 general 3 somewhat 4 very 5}

### 4.2. Factor Analysis of Perceptual Image Semantics

There were 10 to 20 image adjectives initially screened out. If there were too many image vocabularies, it was not conducive to the study and interpretation of user image, and it would increase the cognitive burden of user image evaluation. Therefore, factor analysis was used to extract a few comprehensive image vocabulary to reduce the cognitive dimension. Firstly, the relationship between variables was tested by Kaiser-Meyer-Olkin (KMO) test. The KMO value was closer to 1, the correlation between variables was stronger, and the original variables were suitable for factor analysis. Generally, if the KMO measure was greater than 0.7, and this analysis method could be adopted. Then, Bartlett test of sphericity was used to test the hypothesis that the correlation coefficient matrix was a unit matrix. If the original hypothesis could not be rejected, it could be considered that there was no significant difference between the correlation coefficient matrix and the unit matrix, and then the original variables were not suitable for factor analysis.

### 4.3. The Acquisition and Analysis of Users' Perceptual Image of Cultural and Creative Products

Through Internet, books, and other ways, fifteen adjectives were collected to express the image feelings of cultural and creative products (see Table 4). The samples of flavoring bottle were collected and sorted, and the modeling board with 32 styles of flavoring bottle was established based on the theory of scenario board. These flavoring bottles had obvious differences in shape and style; the purpose was to expand the target users' perception for the shape of bottles.

As mentioned in Section 4.1, the five-point scale was used as a reference for users to quantify the perceptual image to each vocabulary. The questionnaire was conducted to investigate the users. The scale value was set between 1 and 5, and the larger the value was, the closer the sensation of perceptual image vocabulary was.

The experimental results were analyzed by SPSS statistics software 19.0. First by calculating the correlation coefficient matrix, anti-image correlation matrix, Bartlett's test of sphericity, and Kaiser-Meyer-Olkin (KMO) test, the relationship between variables was tested. The statistics observed value was 341.662 in Bartlett's test of sphericity; since the corresponding probability of *P* value was close to 0, less than the significance level of *α* (*α* equal to 0.05), it could be regarded as that there was significant difference between the correlation coefficient matrix and unit matrix. The value of KMO was 0.728; according to the KMO metrics provided by Kaiser, the original variables were suitable to conduct factor analysis.

Four perceptual image semantic factors have been extracted by the principal component analysis method. The cumulative variance contribution rate is 79.348% (see Table 5).

In Table 6, the data shown in boldface of each column represent a higher load on four factors, respectively. For example, the first factor mainly explains the classical, fashionable, and five perceptual image vocabularies. Therefore, the first factor mainly describes the overall style type of cultural and creative products, the second factor mainly reflects users' requirements for interesting and personalized design, the third factor mainly explains users' expectations for the quality design of cultural and creative products' details, and the fourth factor mainly reflects users' consumption tendency.

Based on the above analysis, the vocabularies with high load factor value were selected from each group to define users' image demand for cultural and creative products: distinctive style, novel and interesting, exquisite texture, practical, and unadorned. In this way, the dimension of fifteen variables was reduced to four factors, and most information of the original variables could be reflected.

## 5. Evolutionary Design Based on IGA and BPNN

### 5.1. Construction of Morphological Characteristics

In the construction of evolutionary design framework of cultural and creative product, the shape of flavoring bottle was taken as the research object. And the shape elements of flavoring bottle were decomposed into bottle cap, bottle body, and decorative pattern.

The origin of cultural image was national costume that selected by the EEG experiment in Section 3. Therefore, Yi nationality's costume was set as the inspiration for designer to deconstruct the morphological characteristics of flavoring bottle. The characteristics of Yi nationality's costume are shown in Table 7.

The most representative morphological characteristic of Yi nationality's costume is the headdress for both men and women. They wear upper outer garment with buttons on the right, men wearing trousers and women wearing pleated skirt. Moreover, the Yi people always like to wear the “Cha er wa.” Most of the costume patterns come from the worship of the Yi people, which can be roughly divided into plants, animals, natural phenomena, social life, and geometric patterns. We invited designers to extract the characteristics of Yi nationality's costumes from Table 7 and apply them to the shape design of flavoring bottle. Eight cap features, eight body features, and eight decorative patterns were obtained (see Table 8).

Any complex product form was composed of three basic elements, point, line, and surface, which formed the morphological characteristics of flavoring bottle. According to the morphological method, the flavoring bottle was divided into three types of morphological element units according to its constituent elements, and multiple choices were provided in each type (eight choices were taken as examples in this paper). Combining all the choices, the optimal combination scheme could be selected among the numerous overall schemes. A morphological design model *X* was constructed.

(4) $$\begin{array}{c} X=\left[\begin{array}{cccc} X_{11} & X_{12} & \cdots & X_{1n}\\ X_{21} & X_{22} & \cdots & X_{2n}\\ \vdots & \vdots & \vdots & \vdots \\ X_{m1} & X_{m2} & \cdots & X_{mn} \end{array}\right], \end{array}$$

where *m* represents the number of morphological element unit and *n* represents the number of morphological characteristics in each morphological element unit.

### 5.2. Shape Evolution Based on IGA

Using the optimization ability of genetic algorithm, the morphological characteristics of flavoring bottle were coded to cross, mutate, and select, so as to get the flavoring bottle shape preferred by users. Through the interactive evaluation by users for perceptual image vocabulary, the fitness value of the individual was obtained. The product shape evolution was conducted as follows.

#### 5.2.1. Encoding and Decoding of Morphological Characteristics

There are many forms of encoding. This paper adopted the most widely used binary encoding method proposed by Holland. In the encoding process, binary encoding was carried out for the morphological characteristics in each morphological element unit *X*_*ij*_, and the encoding sequence was bottle cap, bottle body, and decorative pattern. Each morphological element unit had eight morphological characteristics, so it was encoded in a 4-bit binary system with chromosome length of 4 ∗ 3 = 12 bits. Six chromosomes (schemes) were generated by random combination of the system. The initial population of randomly generated flavoring bottle shape and corresponding codes is shown in Table 9.

#### 5.2.2. Take User Evaluation as Fitness Value

Since it was difficult to describe the image perception by constructing function in traditional genetic algorithm, the users' interactive evaluation of image vocabulary was used as the individual fitness value. Each user had different perceptual cognition of individual evaluation. In order to reduce the error caused by users' undirected and uncertain fitness value evaluation, a certain weight should be given to different perceptual image vocabulary. The variance contribution of the factor reflected its explanatory ability to the total variance of the original variable. The higher the value was, the higher the importance of the corresponding factor was. Therefore, the variance contribution rate obtained from factor analysis in Section 4 would be used as the weight. The fitness function was constructed.

(5) $$\begin{array}{c} \text{Fitness}\;\;\left(X\right)=\overset{n}{\underset{i}{\sum }}w_{i}\;*\;E_{i}\left(X\right), \end{array}$$

where *n* is the number of factors, *w*_*i*_ is the weight of each factor, and *E*_*i*_(*X*) is the users' evaluation value of each factor.

#### 5.2.3. Genetic Algorithm Operation

*(1) Selection*. Using roulette as the method of selection, the higher the fitness value, the higher the probability of being selected, and the more likely to generate a new generation.

*(2) Crossing*. A single-point crossing operation was performed. According to the crossover probability, two chromosomes from previous generations were crossed to produce two next generation chromosomes.

*(3) Variation*. By imitating the mutation mode of an individual in the process of biological evolution, the value on a parent chromosome was replaced by the value of remaining individuals' available value, so as to produce offspring.

### 5.3. BPNN Assists IGA

During the interactive evaluation, the individuals generated by each generation needed to score and the user was easily fatigued. So, BPNN was introduced to simulate the users' interactive evaluation, to solve the fatigue problem of IGA and improve the evolutionary efficiency.

#### 5.3.1. Neural Network Structure

BPNN belongs to forward network, which has the advantages of simple network structure, strong nonlinear approximation ability, fast convergence speed, and global convergence. This matched well with the problem to be solved in this paper, that is, the black box model composed of product morphological characteristics elements and user perceptual image. There was no accurate method to express the functional relationship between them. Therefore, it was a good choice to describe the relationship between product morphological characteristics elements and user perceptual image using neural network algorithm. This paper used feedforward BPNN as simulation evaluation model.

**Figure 9:**
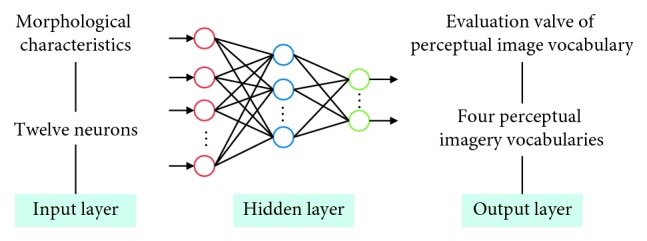
The neural network structure of morphological image evaluation model of flavoring bottle.

The neural network structure of morphological image evaluation model consisted of three layers: input layer, hidden layer, and output layer (see Figure 9). The input layer was the morphological characteristics of flavoring bottle, and the number of input nodes was the number of morphological characteristics of flavoring bottle. The output layer was the perceptive evaluation value of flavoring bottle shape, and the number of nodes was the number of perceptual image adjectives.

*Neurons in input layer*. Each flavoring bottle shape was composed of morphological characteristics in the three morphological element units. The coding of each morphological characteristic was combined in sequence with a total of 12 bits. So, there were twelve neurons in each sample of input layer.

*Neurons in output layer*. The users' perceptual image vocabularies for cultural and creative products were extracted by factor analysis in Section 4, and they were distinctive style, novel and interesting, exquisite texture, practical, and unadorned. The four perceptual image vocabularies were set as target image and the evaluation value as target output value, so the output layer had four neurons.

#### 5.3.2. Hidden Layer Structure and Activation Function

The most commonly used method to determine hidden nodes was the “trial and error method,” that was, setting fewer hidden nodes first, gradually increasing through experiments until the appropriate number was found. Some studies also agreed that when the number of neurons in the hidden layer was half of the total number of the nodes in input layer and output layer, the MSE value was also small. Therefore, this paper set the hidden nodes as *nod*=(*u*+*v*)/2, where *u* and *v* were the number of node in input layer and output layer, respectively, and then through experiments to find the most appropriate number.

In general, the transfer function of hidden layer in BPNN was S-type function, and the output layer was linear function. In this paper, Tan-Sigmoid function was selected as the activation function of hidden layer. The range of this function was (−1, 1), and its function form was as follows:

(6) $$\begin{array}{c} f\left(x\right)=\frac{2}{1+e^{-2x}}-1. \end{array}$$

The linear function Purelin was selected as the activation function of output layer, and its function form was as follows:

(7) $$\begin{array}{c} f\left(x\right)=\left(a\;*\;x\right)+b. \end{array}$$

### 5.4. Algorithm Realization of Shape Evolutionary Design of Cultural and Creative Product

**Figure 10:**
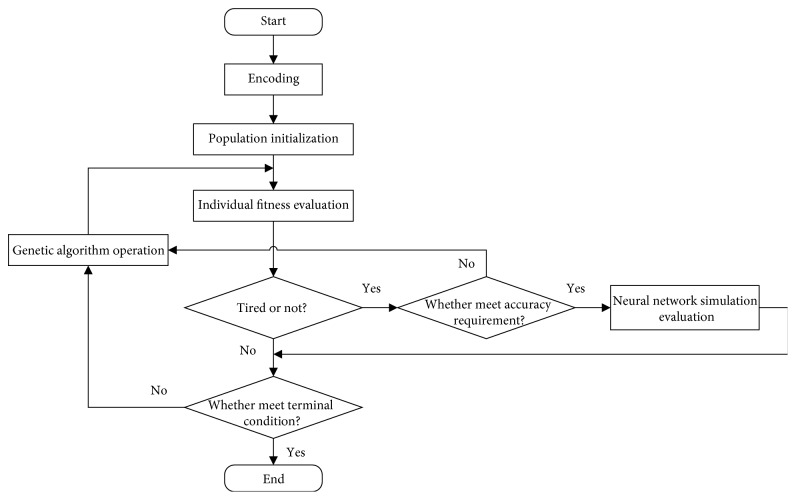
The flow of evolutionary algorithm.

As shown in Figure 10, the implementation steps of the algorithm are as follows:

**Step 1 .**

Start with initial parameters setting. The initial population is generated randomly, and the number of each generation is set as popsize = 6, the search algebra is 50, the crossing rate is 0.7, and the mutation rate is 0.1.

**Step 2 .**

The randomly generated design scheme of flavoring bottle shape is evaluated, and the system begins to train the neural network. If the decision maker feels tired, go to step 4; otherwise, go to step 3.

**Step 3 .**

Determine whether meet the terminal condition of the algorithm. If so, output the optimal solution; otherwise, optimize, and go to step 2.

**Step 4 .**

Determine whether the error meets the accuracy requirement. If it meets the requirement, then simulate the user evaluation through neural network, go to step 3; otherwise; go to step 2.

The terminal conditions of the algorithm are as follows:

**Condition 1 .**

When the user evaluation meets the following formula more than three generations in a row, the interactive genetic process is stopped, and the system outputs the optimal solution of flavoring bottle shape design.

(8) $$\begin{array}{c} \text{Fitness}\left(X\right)\geq \beta *f_{\max}, \end{array}$$

where *β* is the optimal solution factor, the bigger the valve of *β*, the higher the quality of the optimal solution.  *f*_max_ is the maximum evaluation value.

**Condition 2 .**

When the interactive genetic evaluation reaches the preset termination algebra, that is, after 50 generations, the algorithm terminates, and the system outputs the optimal solution of flavoring bottle shape design.

The algorithm framework flow of this system is shown in Figure 10.

## 6. Example of Cultural and Creative Product Design

Based on the design method proposed above, the shape design system of cultural and creative product was constructed, and a case study of flavoring bottle shape design was carried out. In the process of system construction, the neural network toolbox of Matlab was used to train, update, and apply the evaluation model.

### 6.1. System Introduction

Combined with the characteristics of the problem to be solved in this paper, the function type and parameters have been determined.

#### 6.1.1. Training Function

The trainlm () was set as the training function. In Matlab toolbox, trainlm () is a forward network function trained by Levenberg–Marguardt rules, and it is an optimized training algorithm combining the advantages of functions trainbp () and trainbpx ().

#### 6.1.2. Learning Function

The learngdm () was set as the learning function of neural network, and the initial value of the learning rate was set as 0.05. The learngdm () is a gradient descent learning function with additional momentum factors in Matlab, which improves the speed of learning and the reliability of the algorithm by introducing the method of changing weight of momentum factors.

#### 6.1.3. Activation Function

S-type function (Tan-Sigmoid) and linear function (Purelin) were selected as the activation functions of hidden layer and output layer, and their functions were expressed as tansig () and purelin () in Matlab.

#### 6.1.4. Performance Evaluation Parameters

The mean square error (MSE) was set as the performance evaluation function. The function expression was as follows:

(9) $$\begin{array}{c} \text{MSE}=\frac{\sum_{k=1}^{p}{\left(y_{k}-y_{k}^{*}\right)}^{2}}{p}, \end{array}$$

where *y*_*k*_ is the actual output, *y*_*k*_^*∗*^ is the target output, and *p* is the number of neurons in the output layer.

**Figure 11:**
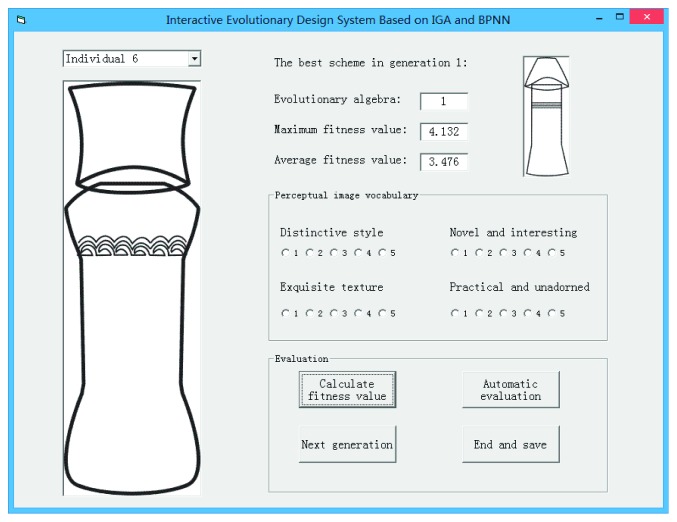
Evolutionary design system.

In this paper, by calling neural network model in Matlab, a shape design system for cultural and creative product was constructed. The system design interface is shown in Figure 11.

### 6.2. Interactive Evaluation Process

For the generated individuals (flavoring bottle schemes), decision maker needed to score each scheme according to five-point scale method, and the system automatically calculated the fitness value of each scheme. As shown in Figure 11, for each scheme, decision maker needs to score four perceptual image vocabularies. Score 1 to 5 indicates the intensity of decision makers' perceptual preference for the scheme, score 1 indicates very weak, and score 5 indicates very strong. In this process, the perceptual preference of decision maker becomes the main factor in the whole evolutionary process. After rating all individuals in a generation, click “calculate fitness value;” the system conducts the genetic evolution of flavoring bottle scheme and decodes the evolution results to present to decision maker. If the evolutionary results are unsatisfactory, the system can continue the evolution process to next generation. In the evaluation process, if the decision maker feels tired, then click “automatic evaluation.” The system will automatically invoke the neural network algorithm in the toolbox to carry out automatic evaluation of flavoring bottle design.

### 6.3. Analysis of Evolutionary Results

#### 6.3.1. The Analysis of Evolutionary Convergence

**Figure 12:**
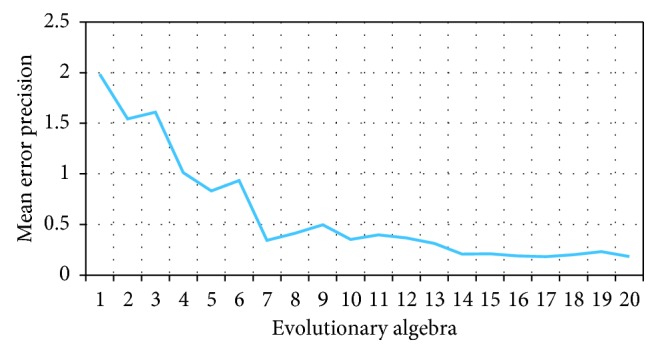
The mean error precision value.

Five users were invited to test the system, and 4.5 points was set as the target fitness value. As shown in Figure 12, the mean error precision is within the range of 0.2 after the 14th generation; in other words, the users' perception for flavoring bottle shape tends to be stable, and it can be evaluated automatically by computer. Continuing with the artificial evaluation of flavoring bottle, it was found that four users have not obtained satisfactory solution after the average 25 generations. In order to reduce the fatigue caused by users' participation in the evaluation, BPNN simulation evaluation was carried out from the 26th generation. After the 40th generation, the evaluation results gradually converged and the evaluation value was greater than 4.5. A satisfactory solution could be found when the user scores more than 4.5 for three consecutive generations.

In the interactive evaluation process, after 14 generations of artificial evaluation, if the user felt fatigued, automatic evaluation could be selected. If the user did not feel tired, they could continue to evaluate the bottle shape until a satisfactory solution was obtained or the maximum number of evaluations was reached.

It could be concluded from the above tests that users could select automatic evaluation after interactive evaluation for 14 generations, and the neural network simulated the remaining evaluations (from the 15th generation to the 40th generation), that is, 14 ∗ 6 = 84 fitness values were evaluated manually, and 26 ∗ 6 = 156 fitness values were evaluated by the neural network. Compared with IGA, IGA integrated with neural network could reduce user fatigue and improve solution quality and speed.

#### 6.3.2. Application Analysis

**Figure 13:**
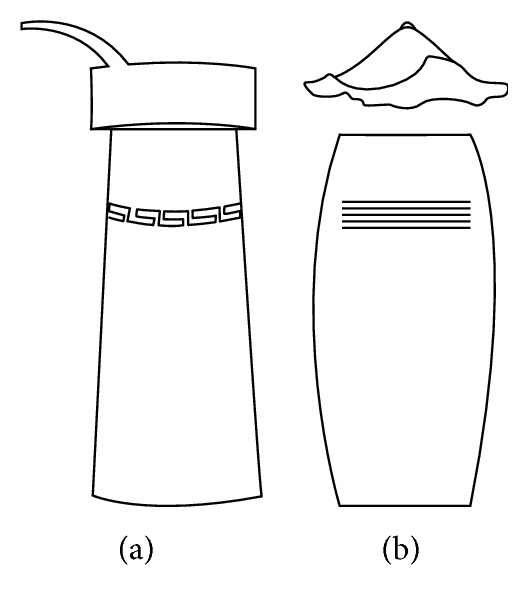
The schemes after system evolution.

**Figure 14:**
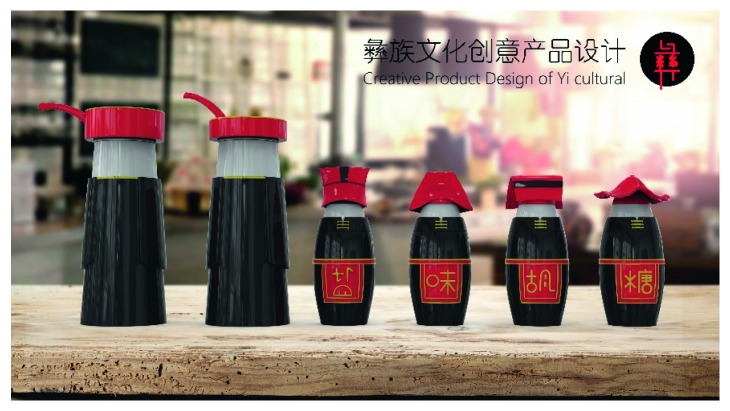
The flavoring bottle design sketch.

A male and a female target user obtained satisfactory solution using this evolutionary system (see Figure 13), and the fitness values were 4.52 and 4.54, respectively. The designer took the schemes in Figure 13 as prototype and designed a set of flavoring bottles (see Figure 14).

**Figure 15:**
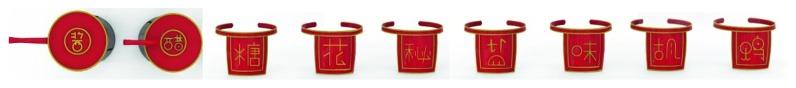
The text design.

**Figure 16:**
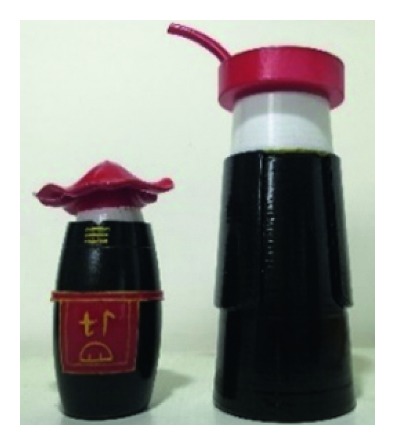
The 3D printing model.

Based on the morphological foundation obtained by the system evolution, the detailed design was carried out. The scheme in Figure 13(a) was used as the prototype of the oil and vinegar bottle. The bottle cap took the hero knot shape of the man's head dress, and the morphological feature of “Cha er wa” was added. The scheme in Figure 13(b) was used as the prototype of flavoring bottle for salt, pepper, and so on. The bottle cap took the shape of lotus leaf from the woman's head dress, and the morphological feature of apron was added. In order to form the serialization, three other bottle caps with the shape of headscarf were designed. In color matching, black, red, yellow, and white were extracted from the color gene of Yi nationality's costume, and the Yi nationality's aesthetic standard of “take black as nobleness” was fully considered. The overall line of the bottle was simple and fluent, and only simple geometric patterns were used for decoration. It conveyed the simple and dignified charm of Yi nationality's costume semantics (see Figure 14). In order to identify the seasoning in the bottle, the text design was made (Figure 15). This series of flavoring bottle design with Yi culture characteristics won the second prize in 2016 China universities industrial design competition (Sichuan competition area). The 3D printing model is shown in Figure 16.

## 7. Discussion

The method proposed in this paper can help designers to design cultural and creative products that conform to the users' target image. However, there are still some shortcomings in this study.

1. The feasibility of the method was verified by taking the flavoring bottle design as an example. In the future, more cultural products need to be refined, and models of all kinds of household products should be established for designers to reference in creative and cultural product design.
2. The relationship between the characteristics in frequency domain and the degree of pleasure under the stimulus of cultural elements with different forms was explored, which only analyzed the characteristics in frequency domain, not involved in the time domain feature, amplitude, and composition and did not consider the effect of color factor and culture factor. The next step of research is to dig deeper into this aspect.
3. As an exploratory study, this paper used a relatively basic interactive genetic algorithm and neural network algorithm to verify the feasibility of the system. In the future, we need to compare the evolutionary effect of different algorithms in the cultural and creative product design and further optimize the algorithm to improve system efficiency and product design quality.
4. The disassembly of flavoring bottle shape was relatively simple, and its morphological characteristics were not very rich. In the future research, we can establish a gene bank of morphological characteristics of cultural and creative products, which is conducive to the evolution of better products.
5. The current system generated two-dimensional wireframe pattern. In the follow-up work, the design scheme can be three-dimensional by adding other programs and tools.

## 8. Conclusion

1. The result of EEG experiment showed that the frontal *α* wave could reflect the relationship between product experience and user emotion, and the frontal alpha asymmetry could be used to judge the pleasure degree of the subjects when facing different cultural elements. Through this method, designers could avoid subjectivity in the selection of cultural elements and relative objectively select the elements of user preferences.
2. Through the user perceptual image investigation, we have realized the research on the perceptual positioning of cultural and creative products. To quantitatively analyze the degree of user preference for the image semantics and master the image feelings of the users for the cultural and creative products, which is conducive to further product shape design and optimization.
3. Based on the cultural elements obtained from the EEG experiment, the morphological characteristics were coded to crossed, mutated, and selected, and the individual fitness value was obtained through the users' interactive evaluation for perceptual image vocabulary, so as to conduct product shape evolution. In order to increase the evolutionary efficiency and improve the fatigue error caused by IGA, BPNN was introduced to simulate users' interaction evaluation. Finally, the evolutionary design system was constructed, and the proposed method was verified by the example of flavoring bottle design.

## Figures and Tables

**Table 1:** The calculated data of PAD emotion scale.

Picture number	Pleasure (*P*)	Arousal (*A*)	Dominance (*D*)	Category of emotional state
1	2.750	2.375	0.255	Type 1
2	−2.175	2.825	1.750	Type 8
3	1.750	1.750	0.015	Type 1
4	1.825	−1.250	0.250	Type 5
5	−2.375	3.015	1.875	Type 8
6	3.000	2.750	2.125	Type 1

**Table 2:** Picture sample groups.

Group	The sample number	The value of pleasure range
Like	1, 6	[2, 4]
General	3, 4	(−2, −2)
Dislike	2, 5	[−4, −2]

**Table 3:** The power ratio of *α* wave in left and right front pole stimulated by six pictures.

Picture number	*F*_P1_	*F*_P2_	*F*_P1_/*F*_P2_	Attribute
1	3.621	4.293	0.84	Like
2	4.028	3.527	1.14	Dislike
3	3.148	3.18	0.99	General
4	3.491	3.678	0.95	General
5	3.648	2.962	1.23	Dislike
6	3.778	5.135	0.74	Like

**Table 4:** Image vocabulary.

1	Unadorned
2	Fashionable
3	Succinct
4	Modern
5	Interesting
6	Elegant
7	Novel
8	Delicate
9	Fluent
10	Practical
11	Classical
12	Attractive
13	Unique
14	Personalized
15	Advanced

**Table 5:** Total variance explained.

Component	Initial eigenvalues			Extraction sums of squared loadings			Rotation sums of squared loadings		
	Total	% of variance	Cumulative %	Total	% of variance	Cumulative %	Total	% of variance	Cumulative %
1	6.215	41.434	41.434	6.215	41.434	41.434	3.678	24.520	24.520
2	2.348	15.651	57.085	2.348	15.651	57.085	2.872	19.144	43.663
3	2.016	13.437	70.522	2.016	13.437	70.522	2.692	17.948	61.611
4	1.324	8.826	79.348	1.324	8.826	79.348	2.660	17.737	**79.348**
5	0.619	4.129	83.477						
6	0.525	3.503	86.980						
7	0.457	3.045	90.025						
8	0.373	2.490	92.514						
9	0.311	2.070	94.585						
10	0.263	1.754	96.339						
11	0.218	1.453	97.791						
12	0.126	0.839	98.630						
13	0.106	0.706	99.336						
14	0.051	0.342	99.678						
15	0.048	0.322	100.000						

**Table 6:** Rotated component matrix.

	Component			
	1	2	3	4
Classical	−**0.892**	−0.129	−0.167	−0.098
Fashionable	**0.686**	−0.205	0.551	−0.012
Succinct	**0.684**	−0.304	0.211	0.377
Modern	**0.677**	−0.140	0.456	0.019
Fluent	**0.654**	−0.173	0.300	0.205
Novel	−0.114	**0.912**	−0.167	0.021
Interesting	0.121	**0.764**	−0.373	−0.110
Personalized	−0.430	**0.715**	0.106	−0.136
Unique	−0.648	**0.650**	0.143	0.028
Advanced	0.219	−0.135	**0.908**	0.199
Delicate	0.294	−0.008	**0.844**	−0.079
Elegant	0.503	−0.402	**0.518**	0.107
Practical	0.054	−0.223	0.009	**0.926**
Unadorned	0.135	−0.150	0.001	**0.924**
Attractive	0.132	0.264	0.118	**0.816**

**Table 7:** The characteristics of Yi nationality's costume.

Headdress	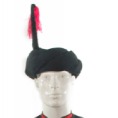	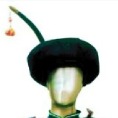	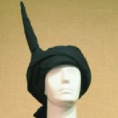	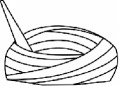
	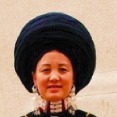	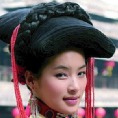	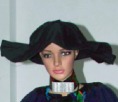	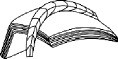

Both men's and women's upper outer garment with buttons on the right	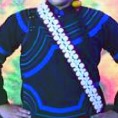	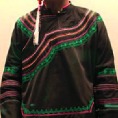	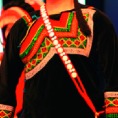	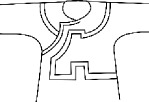
	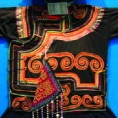	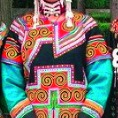	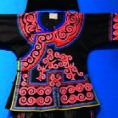	

Men with trousers	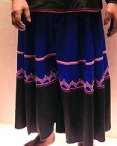	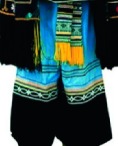	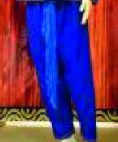	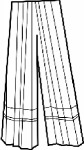

Women with pleated skirts	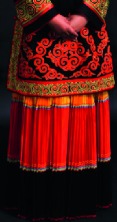	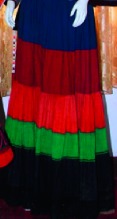	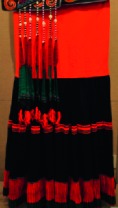	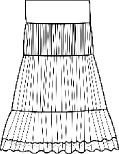

Dressed in “Cha er wa”	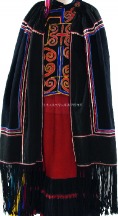	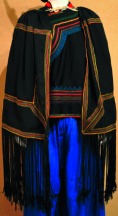	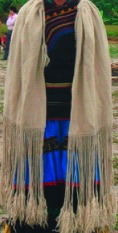	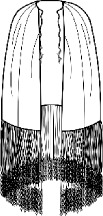

**Table 8:** The morphological characteristics of flavoring bottle.

Bottle cap	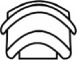	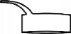	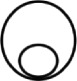	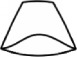	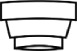	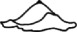	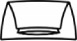	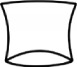

Bottle body	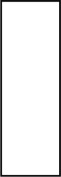	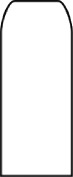	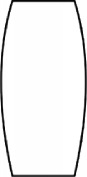	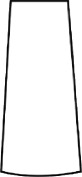	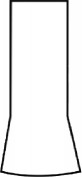	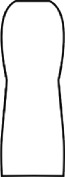	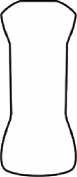	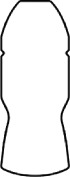

Decorative pattern	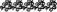	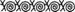	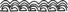	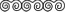	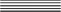	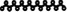	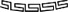	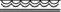

**Table 9:** The initial population of flavoring bottle shape.

*P*_1_	*P*_2_	*P*_3_

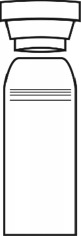	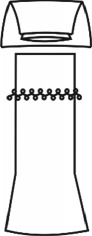	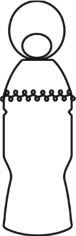
010100100101	011101010110	001110000110

*P*_4_	*P*_5_	*P*_6_

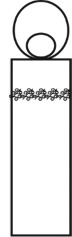	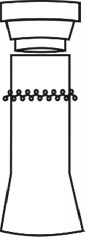	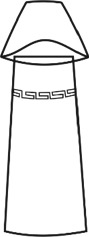
001100010001	010101010110	010001000111

## Data Availability

The data used to support the findings of this study are included within the article.
